# Water, sanitation, and hygiene as a priority intervention for stunting in under-five children in northwest Ethiopia: a community-based cross-sectional study

**DOI:** 10.1186/s13052-021-01128-y

**Published:** 2021-08-24

**Authors:** Ayechew Ademas, Metadel Adane, Awoke Keleb, Gete Berihun, Getu Tesfaw

**Affiliations:** 1 Department of Environmental Health, College of Medicine and Health Science, Wollo University, Dessie, Ethiopia; 2 Department of Pharmacy, College of Health Science, Debre Tabor University, Debre Tabor, Ethiopia

**Keywords:** Associated factors, Children, Ethiopia, Stunting, Under-five, WASH

## Abstract

**Background:**

Stunting was a significant public health problem for under-five in developing countries including Ethiopia. Globally, it was estimated 21.9% or 149 million (81.7 million in Asia and 58.8 million in Africa) under-five children stunted in 2018. In East Africa, 24 million are stunted which is the highest-burden from African regions. Hence, this study aimed to assess the prevalence of stunting and its association with Water Sanitation and Hygiene (WASH) in northwestern Ethiopia.

**Method:**

A community-based cross-sectional study design was conducted among 630 participants from December to mid-January 2019. From five kebeles, two were selected by a simple random sampling technique for the study. To reach study participants a systematic sampling technique was used. Data were collected by using an observational checklist, pretested questionnaire, and anthropometric measurement. Anthropometric indicator, height-for-age was determined using the current World Health Organization (WHO) growth standards. Multivariable logistic regression analysis was computed to analyze the data. From the multivariable analysis the Adjusted Odds Ratio (AOR) with 95% Confidence Interval (CI) and *P-value* < 0.05 were used to declare statistical significance.

**Result:**

The prevalence of stunting among under-five children was 35.6% (95%CI; 31.9–39.5%). The result from this study showed that having illiterate father and mother, give birth before marriage (single), large family size, short maternal height, unimproved drinking water source, unimproved sanitation, poor hygienic practice, having diarrhea in the previous 2 weeks before the data collection, method of child feeding, age at which complementary feeding started, frequency of feeding, not deworming and mothers who had antenatal care visit of fewer than three times were statistically associated with stunting.

**Conclusion:**

In this study, stunting was an important public health problem among under-five children. It remains the same as the national average prevalence of Ethiopia. To alleviate this problem proper family planning utilization, good dietary intake, maternal and paternal education, and WASH interventions are critical.

## Introduction

Stunting is long-lasting malnutrition that happens when kids fail to achieve their linear growth potential. It results in irreversible cognitive and physical damage. A child is ‘stunted’ if the height of the child is less than minus two standard deviations(− 2 SD) of the WHO standard [1]. The process of becoming stunted typically begins in utero, though a child may not be classified as ‘stunted’ before 2 to 3 years of age [2].

Globally, an estimated 21.9% or 149 million (81.7 million in Asia and 58.8 million in Africa) under-five children were affected. It indicates that one in every four was stunted. In East Africa, 24 million children were stunted in 2018 [3]. The intervention did on intrauterine growth restriction, stunting, and severe wasting reduces the number of global deaths and disability-adjusted life years (DALYs) in under-five children [4]. It was reported that stunted children are more to drop out of school and take an extra 1.5 years to achieve and complete their education. Of all school year repetitions, 18% are associated with stunting [5].

Growth failure arises from complex environmental, social, and biological causes that are interlinked at various levels [6]. Denial of safe WASH access is an essential cause of maternal and child health and nutrition problems. The effect was magnificent during the first 1000 days of a child’s life from conception to 2 years of age. Five or more episodes of diarrhea during the 2 years of age contribute to a quarter of stunting in children globally [7]. In 2016, the prevalence of stunting in Ethiopia was 38%. In Amhara regional state the prevalence of stunting was also 46% [8]. WASH is the basic cause of stunting. Poor sanitation alone was the second leading cause worldwide [9]. Globally, 2.2 billion (one in three) people lack safe drinking water. Similarly, 4.2 billion people (3 in 5) population lack safe sanitation and about 673 million people around the world still practice open defecation. Also, 3 billion (2 out of 5) people around the world lack basic handwashing facilities. In Ethiopia, the proportion of the population practicing open defecation was 33%. About 90% of people live in communities where one household practices open defecation. There are also significant inequalities in water service levels between urban and rural populations. For instance, in Ethiopia, there is a 67% gap between rural (5%) and urban (72%) [10].

Poor Sanitation and unsafe drinking water cause diarrheal disease and environmental enteropathy. These inhibit nutrient absorption in the small intestine, which can lead to undernutrition and stunting. WASH interventions can prevent 860,000 Child deaths from undernutrition a year globally [7] and decreased stunting prevalence by 12% in Ethiopia [11].

The long-term effects of stunting on humans are poor cognitive development, school achievement, and economic productivity. Because of its magnitude and overwhelming consequences stunting gets the focus of international attention to reduce by 40% by 2025. With the current trends by 2025, about 127 million under-five children will be stunted. Investment and action are necessary to meet the 2025 World Health Assembly (WHA) target of reducing stunting to 100 million [12].

The government of Ethiopia was implementing WASH to combat malnutrition by organizing as part of the National Nutrition Programme II [13] and ONE WASH national program [14]. To come up and meet this target, many studies have been done to determine the magnitude and associated factors. Despite there were studies that show the linkages of nutrition and stunting, there is a paucity of evidence on environmental factors particularly WASH and stunting. Therefore, this study aimed to determine the prevalence of stunting and association between stunting and WASH factors that were understudied in Ethiopia.

## Methods and materials

### Study design, period, and setting

A community-based cross-sectional study was employed. The study was conducted in Debre Tabor town from December to mid-January 2019. Debre Tabor town is the Zonal Administration center of South Gondar Zone, located 666 km North-West from the capital city of Ethiopia, Addis Ababa. It is found 103 km east away from Bahir Dar, the town of Amhara Regional State, and 50 km East of Lake Tana. The town consists of 5 kebeles (the smallest Administrative unit) in Ethiopia. The total population in 2019/2020 was 89,587. Of all populations, 44,659 are male and 44,928 females [15].

### Source population and inclusion criteria

The source population for the study includes households in Debre Tabor town. The study populations were households selected in the two kebeles. The study unit is all under-five children in the selected households. Under-five children with mothers/caregivers who lived a minimum of 2 weeks in the study area were included in the study. Under-five children with visible deformity and unknown age were excluded from the study.

### Sample size determination and sampling procedure

Sample size was estimated by using a single population proportion formula taking a prevalence of stunting in the Amhara region (46%) [8]. A design effect1.5 was used due to multistage sampling and 10% non-response was added to get a total of 630 study participants. Since we used a two-stage sampling technique subjects from the source population may have unequal selection probabilities resulting in a misleading result. For this reason, we used a design effect for determining effective sample size and to get the true indication of the source population which was supported by literature [16].

To ensure sample size sufficiency, sample size was calculated using the prevalence of stunting (46%) [8] which was 382, calculated using a factor of unsafe water source which was a significant predictor of stunting [17] which was 320, and also the sample size was calculated using combined WASH variables which were significant predictors of stunting [18] which was 204. However, the total sample size calculated using all others was less than the one calculated using the prevalence of stunting.

A multistage stage sampling technique was employed. In the first stage, from 5 kebeles, 2 kebeles were selected by simple lottery method. According to the size of under-five children, sample size was distributed to each kebele proportionally. In the second stage, study participants (households with at least one under-five children) were selected using systematic sampling technique by preparing sampling interval (*N*/*n* = 8) using the sampling frame obtained from the list of households with under-five children in each kebele family folder (family registration book by health extension workers at each kebeles). The first household was selected by choosing one random number out of the sampling interval by lottery method and every 8th household was included until the required sample size was achieved. In cases where there are two or more under-five in the same household, one of them was selected randomly by lottery method.

During data collection children’s mothers or caretakers were interviewed, height/length was measured and observations were made. Unavailable study subjects at the first visit were revisited once more the same day or the following day. Those subjects not available at the second-round visit were considered non-respondent.

### Outcome measurement and explanatory variable

The dependent variable was the prevalence of stunting (height/length-for-age) and independent variables include socio-demographic, WASH, health care, and dietary factors.

### Operational definition

Stunting: is assessed with Height-for-age. It is a measure of linear growth retardation and cumulative growth deficits. Children whose height-for-age Z-score is below minus two standard deviations (− 2 SD) from the median of the reference population are short for their age (stunted), or chronically undernourished. Children who are below minus three standard deviations (− 3 SD) are severely stunted [1].

Pre-lacteal feeding: children given something other than breast milk during the initial 3 days of life.

Complementary feeding: a child receives both breast milk or a breast milk substitute and solid (semi-solid or soft) foods.

Improved sanitation: Are sanitation facilities that hygienically separate human excreta from human contact. It includes latrine facilities that have a connection to a public sewer or a septic system, pour-flush latrine, simple pit latrine with cover/slab, and ventilated improved pit latrine.

An improved water source is a source that, by nature of its construction, adequately protects the water from outside contamination, from fecal matter. It includes piped household connection, public standpipe, borehole, protected dug well, protected spring and rainwater collection.

Good water handling practice: households that store water with a container and covered at the time of visit.

Improved hygienic practice: households that have handwashing facilities with the availability of soap and other detergents near the toilet facility [19].

Diarrhea: is defined as the passage of three or more loose or liquid stools per day (or more frequent passage than is normal for the individual) [20].

### Data collection and quality assurance

The data was collected using a structured questionnaire adapted from WHO Conceptual framework [21] through face-to-face interviews, direct observation, and using anthropometric measurements. The questionnaire comprised of socio-demographic, WASH, health care, and dietary variables. To ensure the consistency of the questionnaire, it was developed in English and translated into Amharic (local language), and translated back into English. Four female BSc degree nurses and two BSc degree environmental health professionals were employed as data collectors and supervisors respectively. To ensure the acquisition of reliable data trained environmental health professionals were employed as supervisors. Data collectors and supervisors were trained by the principal investigator for 2 days on the objectives of the study, the content of the questionnaire, ethical issues, and approaches to be used during data collection. During the training session, a pre-test was done on 32 individuals (5% of the sample size) in non-selected kebele (kebele 03). Then necessary adjustments were made to the questionnaire. The collected data were checked daily by the supervisors and principal investigator for completeness.

The data collector measured and recorded the height/length of children. Double measurements were done to uphold the consistency of anthropometric measurements and an average value with the nearest 0.1 cm was taken. The date of birth for children was collected from the mother or caretaker. For those with written evidence, the date of birth was collected from birth certificates, child health cards, or local event calendars. Length measurement for children 0–23 months was taken in laying down or recumbent position and standing height was taken for children 24–59 months [22].

### Data management and statistical analysis

The collected data were checked, coded, and entered into Epi-Info version 7.2, WHO Anthro and exported to Statistical Package of Social Science (SPSS) version 25.0 for data cleaning and analysis. Both bivariate and multivariable analyses were done. From the bivariate analysis, variables with a *p-value <* 0.25 were retained and enrolled in the multivariable analysis based on different pieces of literature [23–26].

From the multivariable analysis AOR values with a 95% CI and *p-value <* 0.05 were used to declare statistical significance. Model goodness of fitness was checked using Hosmer and Lemeshow test and the *p-value* for the test was 0.11 which suggests a good model (if the *p-value* is *>* 0.05). Multi-collinearity between independent variables was checked using the standard error of the coefficient of the model with a value greater than 2. Collinearity was not observed.

## Results

### Socio-demographic characteristics of participants

Data were collected from a total of 630 mother-child pairs. The mean ages of the study participant were 30.94 months with a standard deviation of 14.597. Of all study participants, 302(47.94%) were males. Regarding the educational level of children’s parents, 160(25.4%) of mothers and 243(38.6%) of fathers were illiterate. About 244(38.73%) children were from households having a family size greater than five. The majority of 514(81.6%) children were born from short (< 150 cm) women. About 41(6.5%), 46(7.3%), and 19(3%) of children’s mothers were single, widowed, and divorced respectively. From the bivariate logistic regression analyses, the marital status of children’s mothers was identified as a determinant of stunting in under-five children (Table 1).

**Table 1:** Bivariate analysis of the association of socio-demographic and economic factors with stunting among children under-five in Debre Tabor town, Ethiopia, December to mid-January 2019

Variables	Frequency	Percent	Stunting		COR(95%CI)	*p-value*
			Yes	No		
Sex of child						
Male	302	47.94	111	191	.904(.652–1.254)	.546
Female	328	52.06	113	215	1	
Educational status of a mother						
Illiterate	160	25.4	43	117	2.066 (1.209–3.53)	.008
Primary level	249	39.5	92	157	1.296(.801–2.095)	.291
Secondary level	126	20.0	48	78	1.234(.717–2.122)	.448
Diploma and above	95	15.1	41	54	1	
Educational status of the father						
Illiterate	244	38.73	74	170	1.875 (1.139–3.089)	.014
Primary level	148	23.50	59	89	1.231(.724–2.096)	.443
Secondary level	149	23.65	51	98	1.569(.916–2.685)	.101
Diploma and above	89	14.12	40	49	1	
Marital status of a mother						
Single	41	6.5	8	33	2.33 (1.053–5.142)	.037
Widowed	46	7.3	19	27	.802(.434–1.481)	.48
Divorced	19	3.0	8	11	.776(.307–1.962)	.592
Married	524	83.2	189	335	1	
Age of mother						
< 20	12	1.9	5	7	.803(.25–2.581)	.712
20–29	264	41.9	90	174	1.108(.794–1.548)	.546
≥ 30	354	56.2	129	225	1	
Family size						
> 5	243	38.6	61	182	2.17 (1.525–3.091)	<.001
≤ 5	387	61.4	163	224	1	
stature of mother						
< 150 cm	116	18.4	26	90	2.17 (1.354–3.474)	.001
≥ 150 cm	514	81.6	198	316	1	
Birth order						
1–2	192	30.5	65	123	1	
3–4	258	40.9	92	166	.923(.624–1.368)	.691
> 4	180	28.6	67	113	.863(.564–1.32)	.498

### Prevalence of stunting

**Fig. 1:**
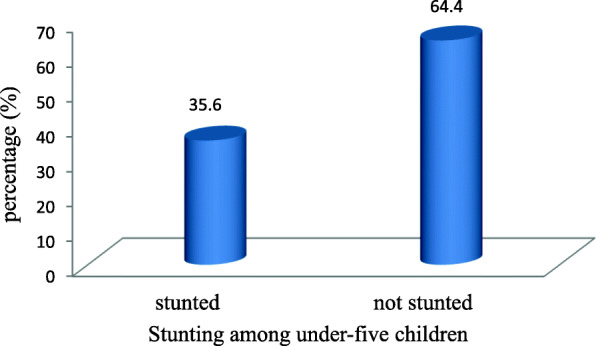
Prevalence of stunting among under-five children in Debre Tabor town Ethiopia, December to mid-January 2019

The prevalence of stunting in under-five children was 35.6 95%CI (31.9–39.5%). Nearly half (17.62%) of stunted children were males and 17.94% were females (Fig. 1).

### Water sanitation and hygiene factors

A latrine was present in all households visited for the data collection. Of all households, 278(44.13%) had an improved toilet facility. About 272(43.2%) households dispose of their solid waste to open fields. The majority of 533(84.6%) households have had an improved drinking water source. About a quarter of 164(26%) of households travel more than 30 min round trip to fetch water, and 544(86.3%) households covered water storage containers (Table 2).

**Table 2:** Bivariate analysis of the association of WASH factors with stunting among children under-five in Debre Tabor town, Ethiopia, December to mid-January 2019

Variables	Frequency	Percent	Stunting		COR(95%CI)	*P*-value
			Yes	No		
Drinking water source status						
Unimproved	97	15.4	14	83	3.854 (2.132–6.97)	<.001
Improved	533	84.6	210	323	1	
Household Water storage container cover						
No	86	13.7	19	67	2.132 (1.245–3.652)	.006
Yes	544	86.3	205	339	1	
Distance to fetch water from the source						
Greater than 30 min	164	26	58	106	1.011(.697–1.467)	.953
Less than 30 min	466	74	166	300	1	
Sanitation status						
Unimproved	352	55.87	106	246	1.712 (1.231–2.379)	.001
Improved	278	44.13	118	160	1	
The hygienic practice of households						
Poor	382	60.6	105	277	2.434 (1.74–3.403)	<.001
Good	248	39.4	119	129	1	
Solid waste disposal						
Open field	272	43.2	79	193	2.363 (1.511–3.695)	<.001
Dump into pit	239	37.9	90	149	1.383(.881–2.17)	.159
Burning	119	18.9	55	64	1	

### Health care factors

About 285(45.2%) children’s mothers had ANC visits to health facilities three and more times before giving birth to the child included in the study. About 296(47%) of children’s mothers also had PNC visits. The majority of 363(57.6%) children had diarrhea in the preceding 2 weeks before the data collection (Table 3).

**Table 3:** Bivariate analysis of the association of health care factors with stunting among children under-five in Debre Tabor town, Ethiopia, December to mid-January 2019

Variables	Frequency	Percent	Stunting		COR(95%CI)	*P*-value
			Yes	No		
Deworming status						
No	288	45.7	87	201	1.544 (1.108–2.151)	.01
Yes	342	54.3	137	205	1	
Diarrhea in the past two weeks						
Yes	363	57.6	103	260	2.092 (1.501–2.915)	<.001
No	267	42.4	121	146	1	
ANC visit of the mother						
< 3times	345	54.8	104	241	1.685 (1.2132.342)	.002
≥ 3	285	45.2	120	165	1	
PNC visit of the mother						
No	334	53	116	218	1.08(.779–1.497)	.646
Yes	296	47	108	188	1	

### Dietary factors

Out of 630 participating children, 388(61.6%) were lactated within 1 h after their birth. Most, 594(94.3%) of children didn’t have pre-lacteal feeding exposure. But, the majority of 582(92.4%) children have lactated the colostrum. About 199(31.59%) of the children’s families feed their children by using their hands whereas 187(29.68%) fed using a cup. From the bivariate analysis, the odds of becoming stunted in children who feed less than three times per day were 3.49 times higher compared to those feeding three times or more (Table 4).

**Table 4:** Bivariate analysis of the association of dietary factors with stunting among children under-five in Debre Tabor town, Ethiopia, December to mid-January 2019

Variables	Frequency	Percent	Stunting		COR(95%CI)	*P*-value
			Yes	No		
Time of initiation of breastfeeding						
After 1 h	242	38.4	83	159	1.094(.781–1.531)	.602
Within 1 h	388	61.6	141	247	1	
Pre lacteal feeding						
No	594	94.3	212	382	1	
Yes	36	5.7	12	24	1.11(.554–2.264)	.774
Colostrum feeding						
No	48	7.6	15	33	.811(.431–1.528)	.517
Yes	582	92.4	209	373	1	
Method of feeding children						
Spoon	134	21.27	51	83	1.75 (1.05–2.919)	.032
Cup	187	29.68	64	123	2.067 (1.278–3.34)	.003
Hand	199	31.59	52	147	3.04 (1.863–4.962)	<.001
Bottle	110	17.46	57	53	1	
Age at introduction of complementary feeding started						
Before 6 and after 8 month	103	16.35	19	84	2.815 (1.66–4.771)	<.001
At 6–8 month	527	83.65	205	322	1	
Frequency of feeding per day						
Twice	174	26.7	33	141	3.49 (2.171–5.616)	<.001
Three times	267	42.3	106	161	1.241(.851–1.810)	.261
> 3 times	189	30.0	85	104	1	

### Factors associated with stunting in under-five children

After adjusting for confounding variables in multivariable logistic regression analysis, our result indicates that; under-five children with illiterate fathers, illiterate mothers, single marital status, a big family size (> 5), short (150 cm) mother, unimproved drinking water source, unimproved sanitation, poor hygienic practice, diarrhea episode in the 2 weeks before the data collection, feeding children by spoon, feeding by cup, feeding by hand, starting complementary feeding before 6 and after 8 months, feeding less than three times per day, lack of deworming treatment and mothers who hand ANC visit of fewer than three times were statistically associated with stunting (Table 5).

**Table 5:** Factors significantly associated with stunting among children under-five from multi-variable logistic regression analysis

Variables	COR(95%CI)	AOR(95%CI)	***P-value (AOR)***
The educational level of the mother			
Illiterate	2.066 (1.209–3.53)	2.572 (1.348–4.909)	.004
Primary level	1.296(.801–2.095)	1.204(.670–2.164)	.534
Secondary level	1.234(.717–2.122)	1.09(.566–2.100)	.797
Diploma and above	1	1	
The educational level of the father			
Illiterate	1.944 (1.176–3.212)	2.275 (1.22–4.242)	.01
Primary level	1.284(.752–2.192)	1.122(.588–2.142)	.726
Secondary level	1.685(.983–2.891)	1.695(.886–3.242)	.111
Diploma and above	1	1	
Marital status			
Single	2.33 (1.053–5.142)	2.717 (1.09–6.772)	.032
Widowed	.802(.434–1.481)	.696(.322–1.507)	.358
Divorced	.776(.307–1.962)	.954(.311–2.922)	.934
Married	1	1	
Family size			
> 5	2.171 (1.525–3.091)	2.255 (1.424–3.571)	.001
≤ 5	1	1	
Stature of mother			
< 150 cm	2.169 (1.354–3.474)	1.776 (1.024–3.081)	.041
≥ 150 cm	1	1	
Source of drinking water			
Unimproved	2.896 (1.689–4.966)	2.413 (1.116–5.219)	.025
Improved	1	1	
Latrine/sanitation status			
Unimproved	3.109 (1.279–7.554)	2.043 (1.359–3.070)	.001
Improved	1	1	
The hygienic practice of households			
Poor	2.434 (1.74–3.403)	1.87 (1.246–2.808)	.003
Good	1	1	
Diarrhea in the past two weeks			
Yes	2.092 (1.501–2.915)	2.904 (1.917–4.399)	<.001
No	1	1	
Deworming status			
Not dewormed	1.544 (1.108–2.151)	1.645 (1.105–2.448)	.014
Dewormed	1	1	
ANC visit of mother			
< 3 times	1.685 (1.2132.342)	2.16 (1.431–3.259)	<.001
≥ 3 times	1	1	
Method of feeding children			
Spoon	1.75 (1.05–2.919)	2.108 (1.126–3.945)	.020
Cup	2.067 (1.278–3.34)	2.154 (1.207–3.843)	.009
Hand	3.04 (1.863–4.962)	3.362 (1.866–6.058)	<.001
Bottle	1	1	
Age at introduction of complementary feeding started			
Before 6 and after 8 month	2.815 (1.66–4.771)	2.215 (1.132–4.337)	.020
At 6–8 month	1	1	
Frequency of feeding per day			
Twice	3.49 (2.171–5.616)	3.542 (1.784–7.034)	<.001
Three times	1.241(.851–1.810)	1.151(.537–2.465)	.718
> 3 times	1	1	

## Discussion

In this community-based cross-sectional study, we estimated the prevalence of stunting and its associated factors in children under five. Our result showed that the prevalence of stunting in under-five children was 35.6% with 95%CI (31.9–39.5%). From all (224) stunted children, the magnitude of stunting doesn’t have a major difference between males and females.

The finding also revealed that under-five children with illiterate fathers and mothers, giving birth before marriage (single marital status), a big family size (> 5), short (< 150 cm) mother, unimproved drinking water source, unimproved sanitation status of the household, the poor hygienic practice of the household, diarrhea episodes in the previous 2 weeks before data collection, method of feeding children, age at introduction of complementary feeding started, less frequently feeding per day, lack of deworming treatment and mothers who had ANC visit of fewer than three times were at a higher odds of stunting.

The prevalence of stunting in this study was almost similar to the national prevalence (38%) of Ethiopia reported by the Ethiopian demographic and health survey (EDHS) 2016, but low as compared to the Amhara national regional state prevalence (46%) [8]. This discrepancy may be due to study time differences. Also, this prevalence was lower than the 2014 prevalence (50.7%) of Ethiopia [27]. A similar study in rural areas of Ethiopia indicates that the prevalence of stunting was 47.5% [28] which was higher than our findings. This difference may be due to study setting, environmental factors, the basic infrastructure of study households, socio-demographic characteristics of children’s parents, and study period.

However, the finding was higher than the prevalence of stunting in Aykel Town which was (28.4%) [29]. This difference may be due to the study subjects’ age category differences and other socio-demographic characteristics. Again the prevalence in this study was consistent with the findings of similar studies in Albuko district, south Wollo Ethiopia (39.3%) [30], in Hossana Town, Southern Ethiopia (35.4%) [31], and Shey Bench district southwest of Ethiopia, (33.3%) [32]. This similarity may be due to the similarity in the study setting and socio-demographic characteristics of study subjects.

In contrast to our study, the sex of the child was a predictor of growth failure in a similar study conducted in Lalibela and West Gojam Zone [33, 34]. In our finding, children whose mothers had no education were at greater risk of becoming stunted and consistent with findings in Hossana, and in 35 Low- and Middle-Income Countries [35, 36]. Another study from the 2014 Zambia demographic and health survey data revealed that children whose mothers had higher education showed 0.25 times less likely to be stunted as compared to children whose mothers had no education [37]. Similarly, paternal education was also a significant predictor of stunting in under-five children which is in line with a result in a systematic review conducted in East Africa [38]. Besides this, the marital status of mothers was also a significant factor in stunting. Children who were born to single mothers before marriage were 2.717 times more likely to become stunted as compared to the others.

In this study, stunting was associated with big family size (> 5). This finding was consistent with the finding from a study conducted in the rural community of Humbo district, Southern Ethiopia [39]. The result also showed that short mother (< 150 cm) was one of the significant factors associated with child stunting which is in line with a finding from a cross-sectional study in 35 Low- and Middle-Income Countries [36]. Another study conducted in Mekelle City, Tigray Region, north Ethiopia also indicates a consistent finding [40].

In our study stunting was associated with unimproved drinking water sources. It was consistent with a finding from a systematic review [38] and a study in Tehuledere District, North-East Ethiopia [17]. However, analysis from surveys in rural India, household access to improved water supply, or piped water was not associated with stunting [41]. An unimproved sanitation facility was significantly associated with stunting in this study. Consistent with our finding, stunting was associated with having no latrine facility [17], and households’ access to toilet facilities was associated with reduced odds of stunting among children in rural India [41]. Another finding from the analysis of Rwanda Demographic and Health Survey 2014/2015 also indicates that the sharing of a toilet was significantly associated with stunting [42].

The poor hygienic practices of households in this study were significantly associated with stunting. However, caregiver’s self-reported practices of handwashing with soap before meals or after defecation were inversely associated in rural India [41]. This may be due to the reporting bias of respondents. But, in our case; the data was collected by direct observation of the presence of a handwashing facility and soap. This study also revealed that stunting was not associated with field disposal of wastes. This finding was contradicting the finding from a study conducted in the rural community of Humbo district, Southern Ethiopia [39].

The literature review also indicated that the combined WASH intervention increased height-for-age Z scores and decreased the risk of stunting by 13% [43]. A result from meta-analysis revealed that WASH interventions were significantly associated with increased pooled mean height-for-age-z- score. This means that children who received combined WASH interventions reduced in growth retardation compared with children who received single interventions [44]. Another operational research project in Ethiopia also indicates that WASH intervention shows a significant increase in mean height-for-age Z-score and a 12.1% decrease in stunting prevalence [11].

Again, finding from our study revealed that stunting was associated with episodes of diarrhea. This was consistent with a finding from Gojam and Mekelle city, Ethiopia [33, 40]. The result was also consistent with a finding in Kersa district, Eastern Ethiopia [45]. In our study deworming status was a significant predictor of stunting which is consistent with a finding in Lalibela town [34]. Results from this study indicate that ANC visits less than three times were significantly associated with stunting. This finding was consistent with a finding from the analysis of Rwanda Demographic and Health Survey 2014/2015 [42].

In our study, the age of introduction of complementary feeding and method of feeding was significantly associated with stunting. This finding was similar to the study finding in West Gojam Zone Ethiopia [33]. Similarly, in a study conducted at Shey bench district, children who had started complementary feeding at less than 6 months or above 6 months were 3.78 times more likely to be affected by stunting than those who started complementary feeding at the age of 6 months [32]. Method of feeding children was affecting children for stunting in which those children who feed by bottle were less likely to become stunted. In addition, the frequency of feeding per day was also a significant predictor of stunting in under-five children.

### Strength and limitation of the study

One of the strengths of this paper is that the WASH variable was directly observed and ascertained by data collectors to reduce self-reporting bias. Having this strength, there is also a potential recall bias from respondents answering questions relating to events happening in the past years before data collection like ANC visits and deworming supplementation. Due to the cross-sectional nature of the study design, it is difficult for establishing cause-and-effect relationships. A longitudinal study with a comparison group may provide better evidence of stunting causation.

### The implication for practice/policy

The findings from this study will imply policymakers and implementers and public health researchers to ascertain factors associated with stunting within the country for policy actions in the fight to reduce it in the study area and other regions of the country. The policy actions should focus on educating the parents, improving the quality of caring practices for children, and ensuring healthy environments through the organization of education within the community, increase access of the community to safe water supply, sanitation, and health care facility.

## Conclusion

In this study, stunting was a major public health problem in children under five. The magnitude was almost the same as national averages, and it requires greater attention. The factors significantly associated with stunting were illiteracy of father and mothers, giving birth before marriage, family size greater than five, short (< 150 cm) mother, unimproved drinking water source, unimproved sanitation, poor hygienic practice, having diarrhea in the previous 2 weeks before data collection period, method of feeding, frequency of feeding, age at which complementary feeding started, not dewormed and mothers who hand ANC visit of (< 3) times. Family planning utilization, maternal and paternal education, Water, Sanitation, and Hygiene (WASH) interventions in all areas of Ethiopia are critical to reducing the level of stunting among under-five children.

## Data Availability

The datasets used and/or analyzed during the current study are available from the corresponding author on reasonable request.

## Abbreviations

ANC: Antenatal care
AOR: Adjusted Odds Ratio
CI: Confidence Interval
COR: Crude Odds Ratio
EDHS: Ethiopian Demographic Health Survey
HAZ: Height-for-age -Z- score
PNC: Postnatal Care
WASH: Water Sanitation and Hygiene
WHO: World Health Organization
